# Supplementary material for: Three-dimensional hidden phase probed by in-plane magnetotransport in kagome metal CsV3Sb5 thin flakes
Source: Nat Commun. 2024 Jun 12;15:5038. doi: 10.1038/s41467-024-49248-3 (PMC11169564; doi:10.1038/s41467-024-49248-3)
Supplement: Supplementary file 1 — Supplementary Information [file 41467_2024_49248_MOESM1_ESM.pdf]

# Supplementary Information for

# Three-dimensional hidden phase probed by in-plane magnetotransport in kagome metal $CsV_3Sb_5$ thin flakes

Xinjian Wei[1†], Congkuan Tian[1,2†], Hang Cui[2], Yuxin Zhai[3], Yongkai Li[4,5,6], Shaobo Liu[1,2], Yuanjun Song[1], Ya Feng[1], Miaoling Huang[1], Zhiwei Wang[4,5,6], Yi Liu[7], Qihua Xiong[1,3], Yugui Yao[4,5,6], X. C. Xie[2,8], Jian-Hao Chen[1,2,8,9*]

[1] Beijing Academy of Quantum Information Sciences, Beijing 100193, China

[2] International Center for Quantum Materials, School of Physics, Peking University, Beijing 100871, China

[3] State Key Laboratory of Low-Dimensional Quantum Physics and Department of Physics, Tsinghua University, Beijing 100084, China

[4] Centre for Quantum Physics, Key Laboratory of Advanced Optoelectronic Quantum Architecture and Measurement, School of Physics, Beijing Institute of Technology, Beijing 100081, China

[5] Beijing Key Lab of Nanophotonics and Ultrafine Optoelectronic Systems, Beijing Institute of Technology, Beijing 100081, China

[6] Material Science Center, Yangtze Delta Region Academy of Beijing Institute of Technology, Jiaxing 314011, China

[7] Center for Advanced Quantum Studies and Department of Physics, Beijing Normal University, Beijing 100875, China

[8] Hefei National Laboratory, Hefei 230088, China

[9] Key Laboratory for the Physics and Chemistry of Nanodevices, Peking University, Beijing 100871, China

**Supplementary Note 1: Crystal structure of $CsV_3Sb_5$ and thickness of the device**

As Supplementary Fig. 1 shows, $CsV_3Sb_5$ (CVS) crystallizes into a hexagonal space group of P6/mmm (No. 191). The crystal has layered structure which consists of alternately stacked alkali-metal Cs layers and V-Sb layers along *c* axis. V atoms form a 2D kagome framework. V atoms and Sb atoms form covalent bonds in in-plane and out-of-plane, and V-Sb layers are then stacked with Cs layers with van der Waals interactions which makes the crystal be easy to cleave between V-Sb layers and Cs layers. The thickness of CVS nanoflake in the main text was measured by atomic force microscope (AFM). After subtracting the thickness of $Al_2O_3$ substrate, the thickness of the CVS nanoflake shown in Figure 1 in the main text was determined to be approximately 32 nm.

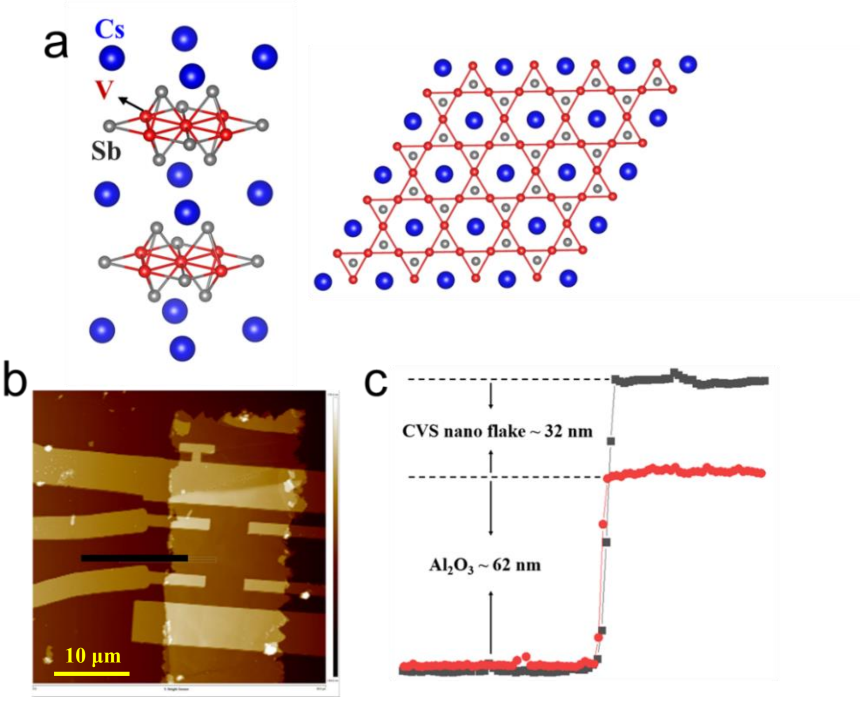


**Supplementary Fig. 1 a,** Crystal structure of $CsV_3Sb_5$. **b,** The AFM micrograph of a $CsV_3Sb_5$ Hall bar device as shown in Figure 1. **c,** Thickness of the black box area of the Hall bar device in **b**. The red curve shows the thickness of $Al_2O_3$ substrate, and the black curve represents the total thickness of $Al_2O_3$ substrate and the $CsV_3Sb_5$ nanoflake.

## Supplementary Note 2: Analysis of SdH oscillations in $CsV_3Sb_5$

Magnetoresistance in the CVS nanoflake exhibits Shubnikov-de Haas (SdH) oscillations at high magnetic field and at low temperature for **B**//z (see Supplementary Fig. 2a**)**. After subtracting the background magnetoresistance, $\Delta R_{xx}$ displays multiple frequencies in 1/**B** as shown in Supplementary Fig. 3a, indicating the contributions of several electron pockets to the oscillations. Supplementary Fig. 3b exhibits the fast Fourier transform (FFT) spectra of the SdH oscillations in Supplementary Fig. 3a, revealing several frequencies at $F_1$=17 T, $F_2$= 28 T, $F_3$= 73 T, and $F_4$=89 T. The FFT amplitudes reduce gradually with increasing temperature. By fitting FFT amplitudes vs. $T$ via the temperature dependence part of the Lifshitz-Kosevich (L-K) formula, we obtained cyclotron effective mass $m^*$=0.083 $m_e$, 0.081 $m_e$, 0.148 $m_e$ and 0.133 $m_e$ for $F_1$, $F_2$, $F_3$ and $F_4$, respectively, where $m_e$ is free electron mass. The fitting formula is $\frac{\eta m^* T/B}{\sinh(\eta m^* T/B)}$,where $\eta = \frac{2\pi^2 k_B m_e}{e\hbar} \approx 14.7\ T/K$ , the average magnetic field **B** = 10 T[1]. Such a small m* is consistent with transport originating from the Dirac modes expected near the M point.

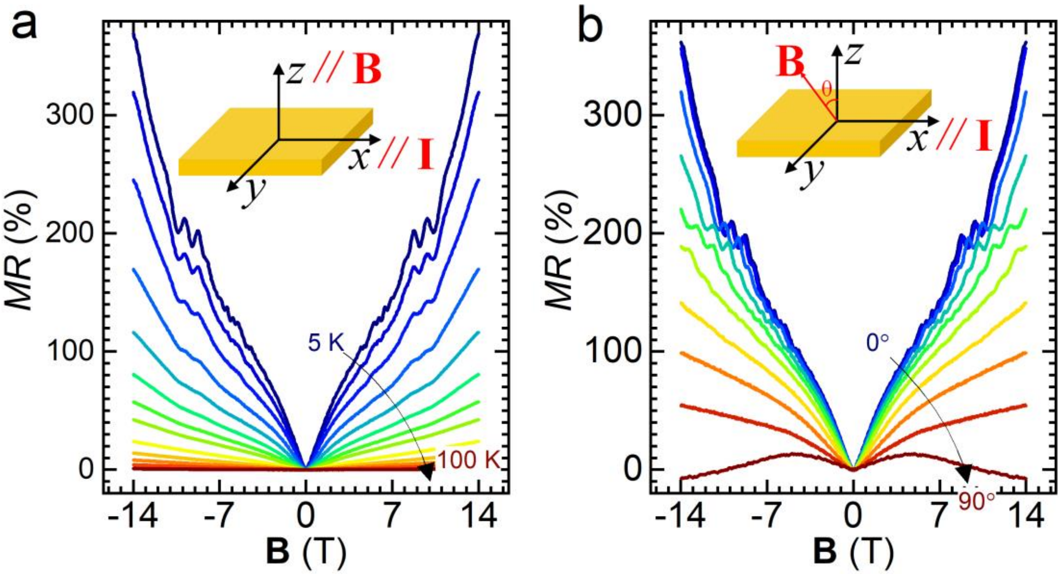


**Supplementary Fig. 2** Shubnikov-de Haas (SdH) oscillations in CVS. **a,** Magnetoresistance vs. perpendicular magnetic field (MR vs. $\mathbf{B}_\perp$) curves for temperatures from 5 K to 100 K. **b,** MR vs. **B** curves for rotating magnetic field directions in the $y$-$z$ plane and at $T$ = 5 K

For $\rho_{xx} \gg \rho_{xy}$ in our data, the Landau index integer $\nu$ corresponds to the maxima of $\rho_{xx}$ while

the Landau index half-integer $\nu + 1/2$ corresponds the minima. We extracted the Landau index $\nu$ vs 1/**B** of frequency $F_3$ at 2 K and plotted it in Supplementary Fig. 3d. Through linear fitting of $\nu$ vs 1/**B**, the intercept on $\nu$ axis is 0.62, corresponding to the phase factor in the L-K formula. The phase factor is $\Gamma$-$\varphi$+$\varsigma$, where $\Gamma$=0.5-$\Phi_B/2\pi$, $\varphi$ is determined by the dimensionality of FS ([$\varphi$=0 and ±0.125 for the respective two-dimensional (2D) and 3D cases]), and $\varsigma$=0.5 ($\rho_{xx} \gg \rho_{yx}$) or 0 ($\rho_{xx} \ll \rho_{yx}$)[1]. In our case, it is suggested that the berry phase $\Phi_B$ is equals π and $\delta$ is close to -0.125, meaning that CVS has a 3D topological non-trivial band structure probably linked to 3D charge order. 3D Fermi surfaces from charge order has also been confirmed in Ref. 2.

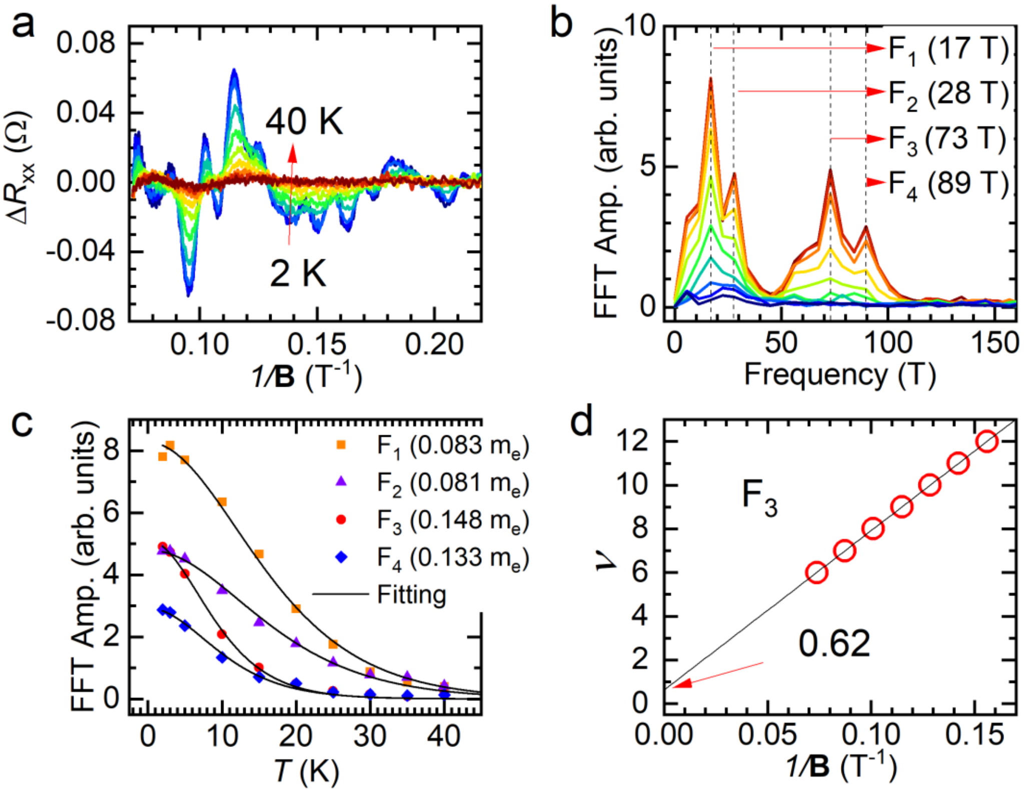


**Supplementary Fig. 3** The analysis of SdH oscillations in CVS nanoflakes. **a,** Oscillatory component $\Delta R_{xx}$ as a function of 1/**B** in the temperature range from 2 K to 40 K. $\Delta R_{xx}$ was extracted from $R_{xx}$ by subtracting a fitting background. **b,** FFT of SdH oscillations in **a**. Four main frequencies $F_1$, $F_2$, $F_3$ and $F_4$ were found. **c,** FFT amplitudes vs. $T$ fitted by the L-K formula, where the scattered symbols are experimental data and the solid lines represent the fitting results. **d,** Landau index $\nu$ vs 1/**B** at 2 K for

$F_3$. The black curve is the linear fitting and the intercept on $\nu$ axis is 0.62.

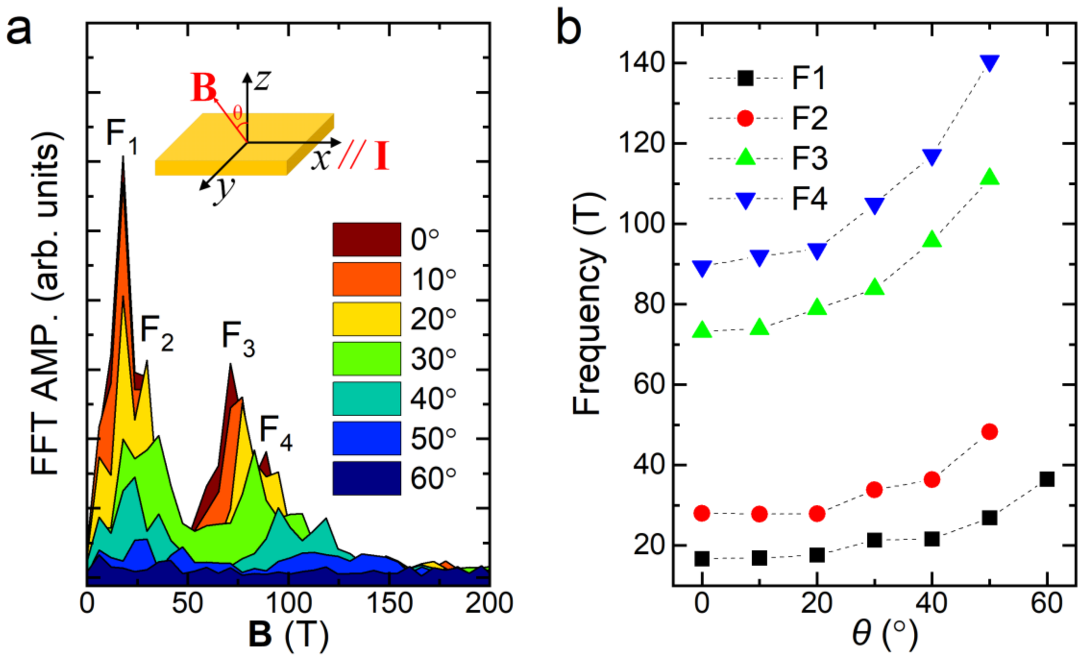


**Supplementary Fig. 4** **a,** Field dependence of FFT amplitudes of SdH oscillations at various field directions ($\theta$s) when $T$ = 5 K. Inset in **a** shows the definition of $\theta$. **b,** Oscillation frequencies ($F_1$, $F_2$, $F_3$ and $F_4$) are shown as a function of rotation angle $\theta$. Due to the broadening and shortening of peaks, Gaussian fits of peaks are used to determine the frequency values.

Furthermore, we have investigated the quantum oscillations with rotating **B** direction, which can provide information regarding the topography of the Fermi surface. We defined the angle between the $z$ axis and **B** as $\theta$. Supplementary Fig. 4a presents a series of FFT amplitudes with different $\theta$. The peak amplitudes for all frequencies obviously decrease with increasing $\theta$, and the peak is barely observed when $\theta$ reaches 60°. In Supplementary Fig. 4b, we plotted oscillation frequencies ($F_1$, $F_2$, $F_3$ and $F_4$) as a function of angle. As $\theta$ increases, $F_3$ and $F_4$ move towards higher frequencies by a large margin, while $F_1$ and $F_2$ have a relatively small increase. This result implies that the Fermi surface has a quasi-2D or prolate ellipsoid shape. This is probably because the interlayer interaction is relatively strong in the CVS.

**Supplementary Note 3: Discussions on positive in-plane magnetoresistance in the low field regime**

In the main text's Fig. 3a and 3b, the in-plane MR initially increases linearly with magnetic field (for **B** < ~4 T), deviates from linearity, reaches a maximum near 6 T, and then gradually decreases. To gain insight into the origin of this positive linear in-plane MR, several possible mechanisms are discussed below:

1) **Superconducting fluctuations:** In cuprate superconductors, the in-plane magnetic field destroys the cooper pairs and inhibits the superconducting fluctuations near $T_c$, resulting in positive in-plane MR[3, 4]. However, in CVS, $T_c$ is ~3 K, and positive in-plane MR remains up to 70 K, beyond the range in which superconducting fluctuations can exist. Therefore, this scenario cannot explain the positive MR in CVS.

2) **Weak anti-localization effect:** Positive in-plane MR at low fields has been observed in topological semimetal $ZrTe_5$[5], TaAs[6] and $Cd_3As_2$[7], which are attributed to weak anti-localization. Positive MR caused by weak anti-localization follows the Hikami-Larkin-Nagaoka formula[8] and exhibits a sharp dip at low magnetic fields, which differs from our observations. Moreover, weak anti-localization is a temperature-sensitive quantum correction to classical conductance typically observable at low magnetic fields ($B$ <1 T), inconsistent with our results.

3) **Guiding center motion of cyclotron orbitals model:** When the mean free path of an electron is much greater than the cyclotron orbit radius $r_c$ under a sufficiently strong magnetic field, and the potential disorder correlation length $\xi >> r_c$, motions of electron cyclotron orbits, known as guiding center motion, dominates the electron trajectories, leading to linear MR dependence on **B** above a turn-on magnetic field. This model explains linear MR in half-metallic $Sr_2CrMoO_6$ thin films[9] and Dirac semimetals $Cd_3As_2$[10]. However, in our case, the magnetic field of the linear MR is below 0.5 T, and at 0.5 T, $r_c$ ~ 384 nm, which is much larger than the sample thickness. Thus, electrons would undergo interface scattering and be unable to form cyclotron motion at low magnetic fields, rendering this model unsuitable for explaining our observations.

4) **Nano-domain scattering:** Wu et al. found that nano-domain boundaries with ripples in trilayer graphene act as a spin filter and generate positive in-plane MR at low temperatures[11]. However, this

MR has a $\mathbf{B}^2$ dependence, unlike our observations. Additionally, forming nano-domain boundaries with ripples in our tens of nanometer thick samples is almost impossible. Thus, this model can also be ruled out.

Since the previously mentioned mechanisms fail to explain the experimental observations, we explore other possibilities. In the analysis of in-plane MR symmetry, we observe that orbital MR caused by the Lorentz force effect leads to a two-fold symmetry ($C2^*$) at low field. This indicates that orbital MR dominates the positive linear in-plane MR. Our ansatz is that in CVS, electron scattering occurs due to impurities, phonons, interface, and charge order domains. The application of a magnetic field changes the electron's orbital path and consequently reduces the electron mean free path, leading to the positive in-plane MR in CVS. The linear behavior in the in-plane MR may be due to the combined effect of different scattering mechanisms. Additionally, we found that the linear MR can be well-fitted by the empirical formula $MR = \mathrm{A}\left[\sqrt{(\mathbf{B}^2 + m^2)} - m\right]$, where $A$ and $m$ are fitting parameters. The fitting results are shown in Supplementary Fig. 5a and 5b.

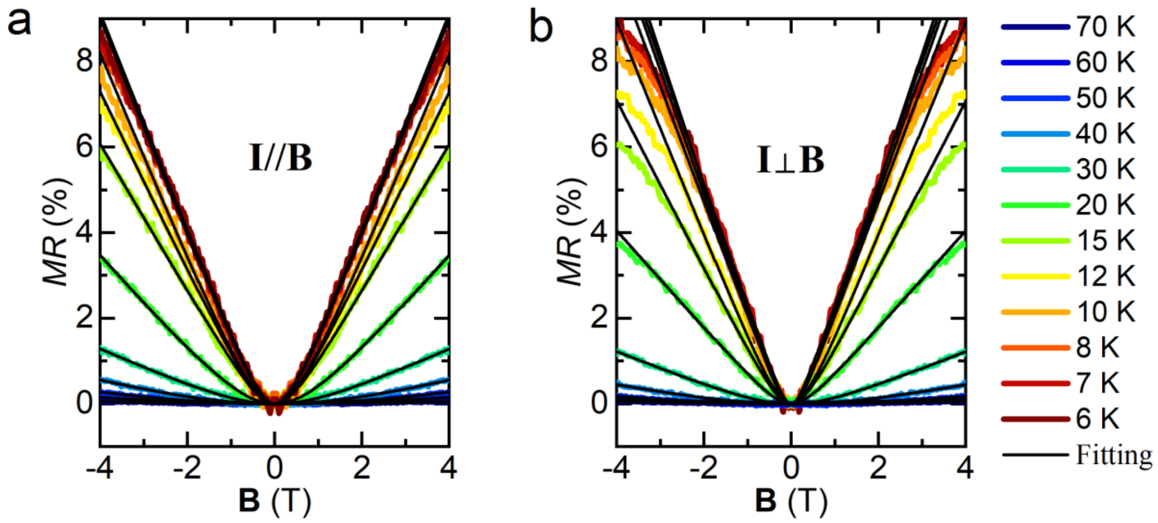


**Supplementary Fig. 5** Fitting results of in-plane magnetoresistance in low field regime. **a,b,** In-plane MR at various $T$ with the **B** parallel to the current direction (**a**) and perpendicular to the current direction (**b**). The solid black lines are fitting curves. The fitting formula is $\mathrm{MR} = \mathrm{A}\left[\sqrt{(\mathbf{B}^2 + m^2)} - m\right]$ where $A$ and $m$ are fitting parameters.

**Supplementary Note 4: Symmetry analysis of in-plane magnetoresistance**

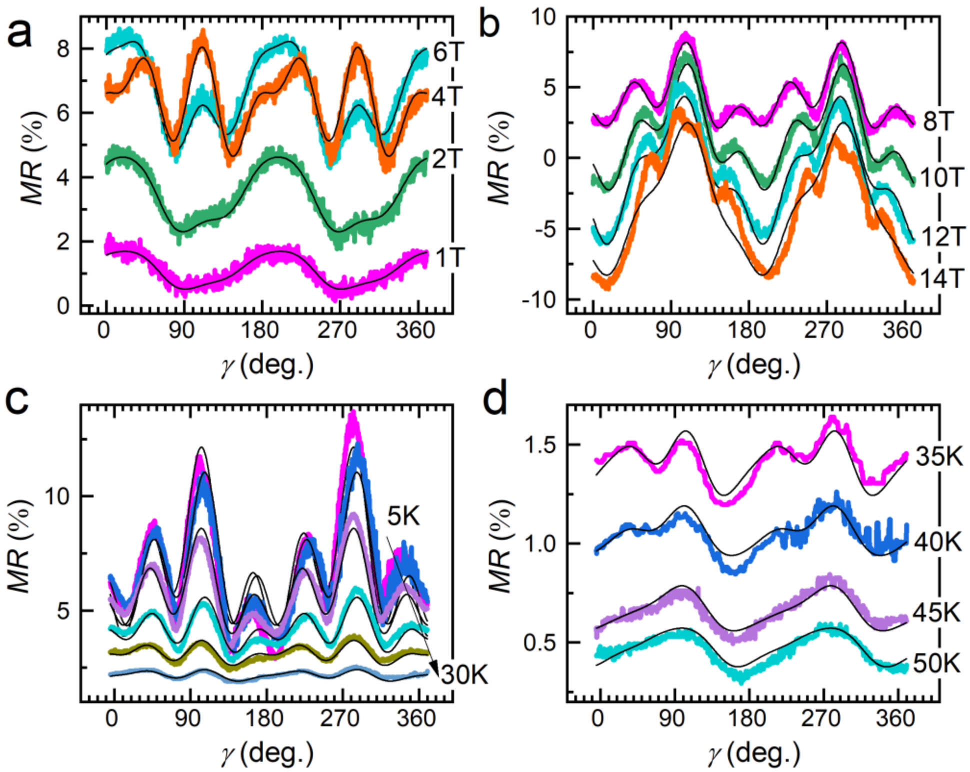


**Supplementary Fig. 6 a,b,** In-plane MR vs angle $\gamma$ with different magnetic field at 5 K. **b,c,** In-plane MR vs $\gamma$ under 9 T magnetic field at different temperatures. The colorful lines are experimental data and the black solid lines are fitting curves. The fitting formula $MR = \alpha + \xi_1 \cos\{(2(\gamma + \eta_1)\} + \xi_2 \cos\{(4(\gamma + \eta_2)\} + \xi_3 \cos\{6(\gamma + \eta_3)\}$, where $\alpha$, $\xi_i$ and $\eta_i$ (i=1,2,3) are the fitting parameters. $\gamma$ = 0 is defined as the magnetic field is perpendicular to the current direction.

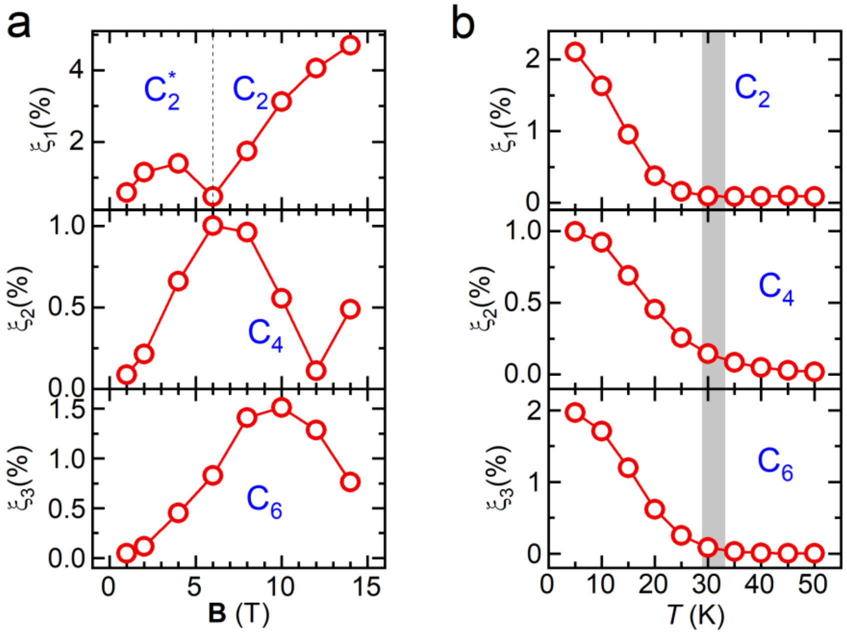


**Supplementary Fig. 7 a,b,** The fitting amplitudes $\xi_i$ from Supplementary Fig. 6 as a function of **B** in **a** and $T$ in **b**.

**Supplementary Note 5: Symmetry of in-plane magnetoresistance in circular disc devices**

When performing in-plane MR measurements, the orbital MR exhibits anisotropic behavior due to the Lorentz Force effect ($\mathbf{F} = q\ \mathbf{v} \times \mathbf{B}$)[12, 13]. The in-plane MR is greater in $\mathbf{B} \perp \mathbf{I}$ configuration than in the $\mathbf{B} /\!/ \mathbf{I}$ configuration. To consider a general electrical conductor, the resistivity tensor in the magnetic field coordinate can be expressed as[14, 15]:

$$\begin{pmatrix} E_{x'} \\ E_{y'} \end{pmatrix} = \begin{pmatrix} \rho_{\parallel} & 0 \\ 0 & \rho_{\perp} \end{pmatrix} \begin{pmatrix} j_{x'} \\ j_{y'} \end{pmatrix}. \qquad (1)$$

where the $x'$ direction corresponds to the $B$-field direction and the $y'$ direction is perpendicular to the field. By using the sample itself as the coordinate system and following a standard coordinate transformation procedure, Eqn. S1 is transformed to:

$$\begin{pmatrix} E_x \\ E_y \end{pmatrix} = \begin{pmatrix} cos\gamma & sin\gamma \\ -sin\gamma & cos\gamma \end{pmatrix} \begin{pmatrix} \rho_{\parallel} & 0 \\ 0 & \rho_{\perp} \end{pmatrix} \begin{pmatrix} cos\gamma & -sin\gamma \\ sin\gamma & cos\gamma \end{pmatrix} \begin{pmatrix} j_x \\ j_y \end{pmatrix}$$

$$= \begin{pmatrix} \rho_{\parallel} cos^2\gamma + \rho_{\perp} sin^2\gamma & (\rho_{\perp} - \rho_{\parallel}) sin\gamma cos\gamma \\ (\rho_{\perp} - \rho_{\parallel}) sin\gamma cos\gamma & \rho_{\parallel} sin^2\gamma + \rho_{\perp} cos^2\gamma \end{pmatrix} \begin{pmatrix} j_x \\ j_y \end{pmatrix}$$

Consequently, $\rho_{xx} = \frac{E_x}{j_x} = \frac{\rho_{\perp} + \rho_{\parallel}}{2} + \frac{\rho_{\perp} - \rho_{\parallel}}{2} cos2\gamma,$ where the $x$ direction aligns with the electric current

and $\gamma$ represents the angle between the $y$-axis and the magnetic fields. Evidently, this Lorentz force-induced orbital MR displays two-fold symmetry, and its orientation is influenced by the direction of the current.

From analysis of in-plane MR symmetry and MR vs **B** curves, we speculated that in-plane MR is mainly attributed to the classical MR at low **B** due to change of electron mean free path by Lorentz force, but at high magnetic field dominated by effect of a certain charge/current order (the so-called "hidden" order). As it is well known, the classical MR caused by the Lorentz force depends on the current direction. To further verify our speculation, we fabricated the circular disc devices with 24 electrodes and measured the in-plane MR with different current directions. The optical image of the device is shown in Supplementary Fig. 8a. The red arrows represent the current directions, and the black arrow denotes magnetic field direction which is at angle $\gamma = 0°$. Supplementary Fig. 8b shows polar representation of the in-plane MR vs. $\gamma$ at 5 K and 1 T with different current directions. It is found that the $C2^*$ symmetric component in the low-field in-plane MR is consistent with a classical MR, where its maximum is perpendicular to the current direction and its direction rotates together with the current direction. However, in the Supplementary Fig. 8c, the C2 symmetry at 12 T has almost no change as the current direction is changed. This data unambiguously proved that the $C2^*$ symmetry under low magnetic field originates from Lorentz force, and under high magnetic field the C2 symmetry stems from the intrinsic twofold symmetry of the electronic states in CVS.

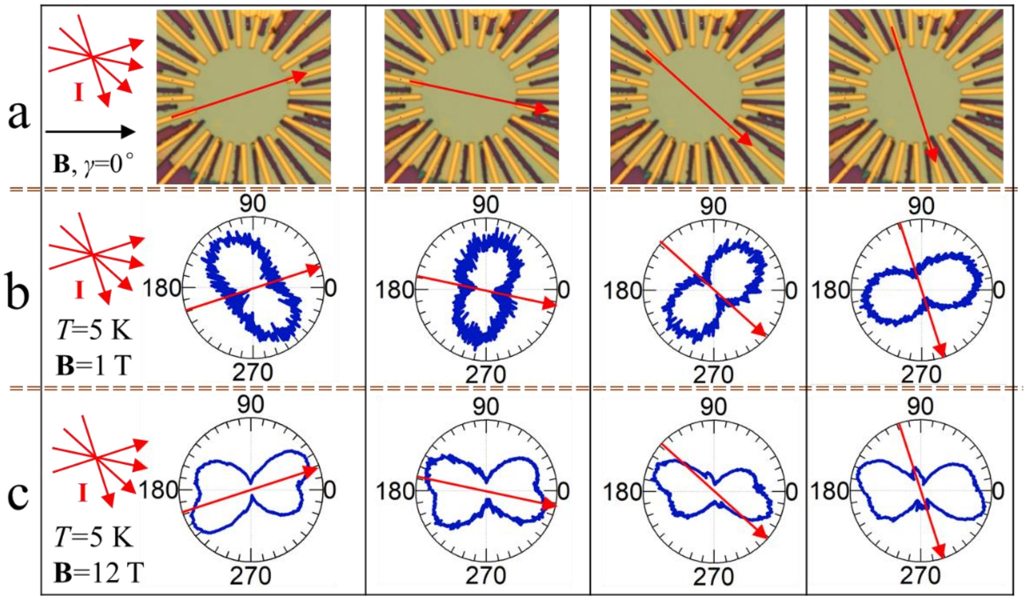


**Supplementary Fig. 8** Magnetoresistance with different current directions in a CVS circular disc device. **a,** The optical image of the circular disc device. The red arrows represent the current directions and the black arrow indicates the direction of the magnetic field with $\gamma$ = 0. **b,** In-plane *MR* in polar representation with 1 T magnetic field for various current directions. **c,** In-plane *MR* in polar representation with 12 T magnetic field. All the data were collected at 5 K.

**Supplementary Note 6: Hall resistance data**

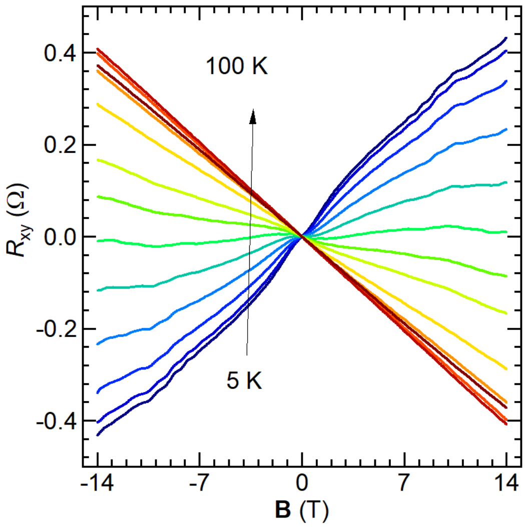


**Supplementary Fig. 9** Hall resistance $R_{xy}$ as a function of magnetic field at different temperatures.

**Supplementary Note 7: Temperature dependent mobility**

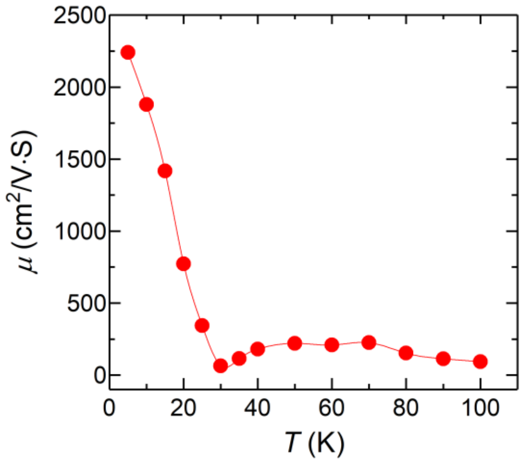


**Supplementary Fig. 10** The mobility $\mu$ as a function of temperature. From Drude model, $\mu=R_H/\rho_{xx}$. Here $R_H$ is the Hall coefficient, the slope of $\rho_{xy}$ vs. **B** under strong magnetic field and $\rho_{xx}$ is the longitudinal resistivity.

**Supplementary Note 8: Reproducibility experiment of hidden phase transition in CVS**

To test the reproducibility of experimental result in the main text, we performed the in-plane magnetoresistance measurement in several devices and observed similar results, one of which was plotted in Supplementary Fig. 11 and 12. With increasing **B**, the symmetry of the in-plane magnetoresistance gradually changes from C2* at low field to C2 plus C6 at high field (Supplementary Fig. 11a). In Supplementary Fig. 12b, the temperature dependence of in-plane MR symmetry displays a similar behavior that the reduction of the C6 component happened at ~30 K with increasing temperatures, suggesting the hidden phase transition. This transition was also characterized by a sudden increase in the negative in-plane MR as shown in Supplementary Fig. 12.

Furthermore, we also measured the in-plane MR symmetry near the CDW transition temperature ($T_{CDW}$~85 K). In Supplementary Fig. 13, the symmetry of the in-plane MR always maintains a two-fold rotational symmetric pattern, while its direction rotates nearly 60° at $T_{CDW}$. Above $T_{CDW}$ (~85 K), the direction of MR minima is parallel to current direction, consistent with C2* direction in Fig. 1c and Supplementary Fig. 8b in which the C2* MR results from Lorentz force effect. Below $T_{CDW}$, CDW

phase leads to an unconventional two-fold rotational symmetric pattern C2.

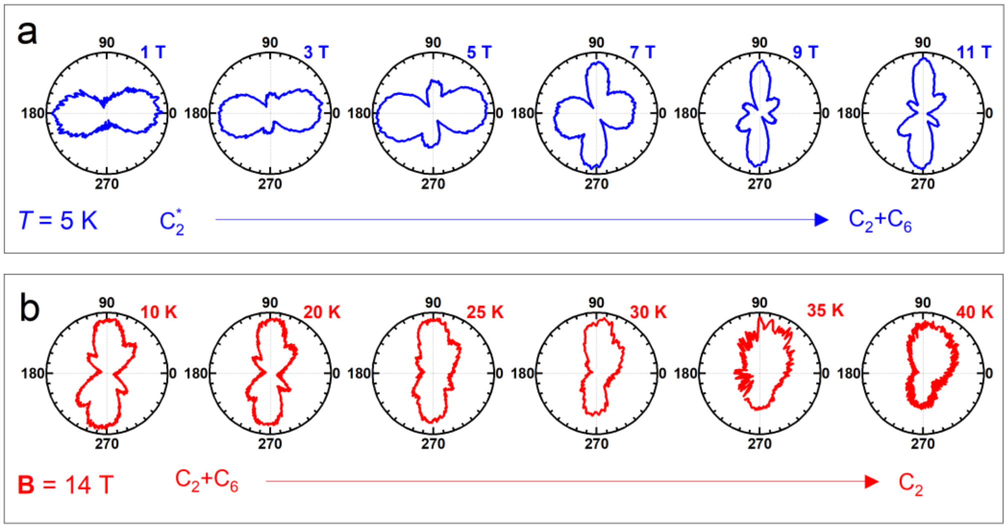


**Supplementary Fig. 11 a,b,** Polar representation of the in-plane MR vs. $\gamma$ at $T$ = 5 K for **B** = 1, 3, 5, 7, 9, 11 T in **a** and at **B** = 14 T for $T$ = 10, 20, 25, ,30, 35, 40 K in **b**. The center point in each polar plot is offset to clearly show the MR anisotropy.

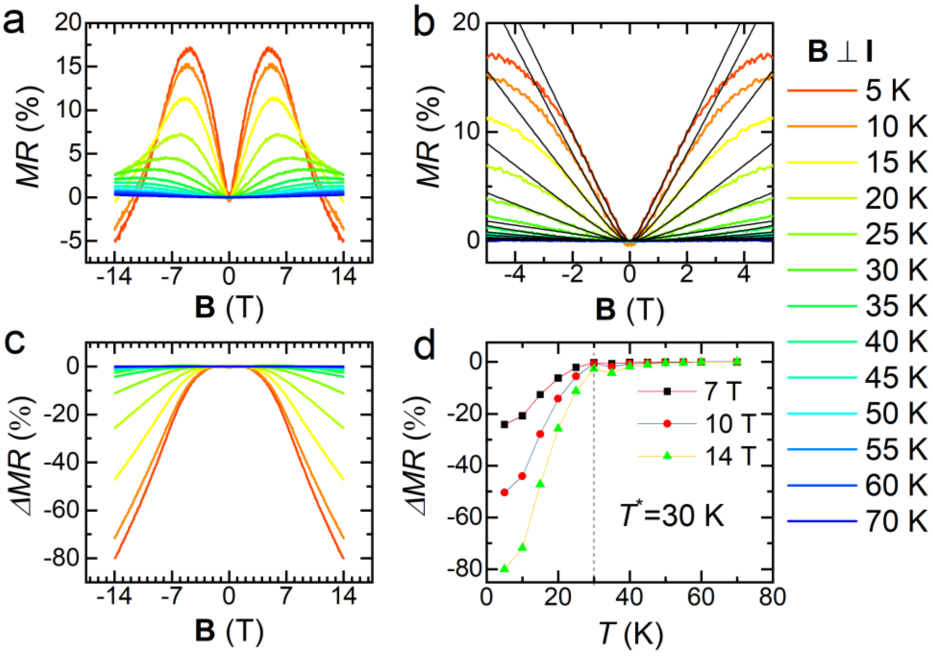


**Supplementary Fig. 12 a,** In-plane MR at various $T$ with the **B** perpendicular to the current direction. **b,** Fitting results of in-plane magnetoresistance in low field regime. The fitting formula is MR = $A\left[\sqrt{(\mathbf{B}^2+m^2)}-m\right]$ where $A$ and $m$ are fitting parameters. **c,** The net negative in-plane

magnetoresistance by subtracting the fitted positive component, i.e. $\Delta MR = MR(\mathbf{B}) - MR_{\text{fitted}}(\mathbf{B})$. **d,** *T* dependent $\Delta MR$ for **B** = 7, 10, 14 T.

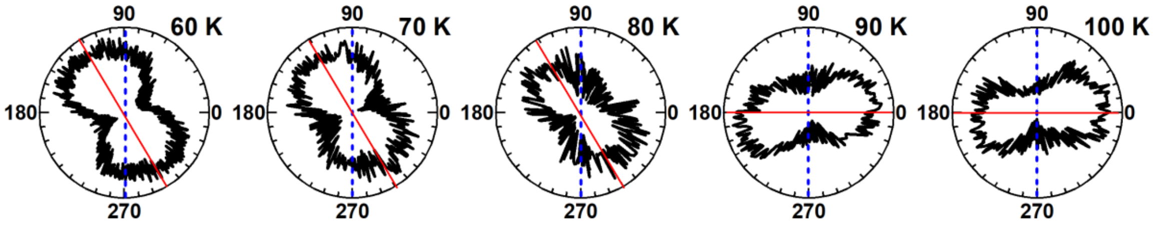


**Supplementary Fig. 13** The variation of in-plane magnetoresistance symmetry near CDW transition temperature ~85 K. Here the current direction is along the 90° direction (marked as blue dotted line) and the red solid lines are a guide to the eye for the maximum direction of the C2 symmetric in-plane MR.

**Supplementary Note 9: Raman scattering measurements**

To determine the orientation of flake device and investigate its possible existence of strain from substrate, we performed the Raman scattering measurements in flake and bulk samples using a $\lambda$=633 nm laser and HORIBA550 spectrometer. The samples were mounted in a vacuum chamber during data acquisition. Temperature control was achieved using a Montana Instrument Cryostation. Polarization-resolved angular-dependent experiments were carried out using a rotatable superachromatic $\lambda/2$ wave plate with collinear polarization configuration. The angle interval is 10° .

In Supplementary Fig. 14b, two prominent peaks at ~119 $cm^{-1}$ and 137 $cm^{-1}$ were detected in both samples, which respectively correspond to the phonon modes $E_{2g}$ and $A_{1g}$, consistent with other Raman studies[16, 17, 18]. The $E_{2g}$ and $A_{1g}$ modes represent the in-plane vibration and the out-of-plane vibration of Sb atoms, respectively. The structure of CVS belongs to $D_{6h}$ point group. As for $D_{6h}$ point group, the $A_{1g}$ and $E_{2g}$ Raman-active modes that contribute to the back-scattering response have the following Raman tensors:

$$R_{A_{1g}} = \begin{pmatrix} a & 0 & 0 \\ 0 & a & 0 \\ 0 & 0 & b \end{pmatrix}; R_{E_{2g}} = \begin{pmatrix} 0 & f & 0 \\ f & 0 & 0 \\ 0 & 0 & 0 \end{pmatrix}, \begin{pmatrix} f & 0 & 0 \\ 0 & -f & 0 \\ 0 & 0 & 0 \end{pmatrix}$$

The Raman scattering intensity of both modes are expected to be independent of angle of the linear polarization, $I_{\mathrm{A1g}} \propto a^2$ and $I_{\mathrm{E2g}} \propto f^2$. However, there are subtle polarization dependencies detected as shown in Supplementary Fig. 14c. According to Ref. 16, this phenomenon may be related to CDW order and the intensities of $E_{2g}$ mode reach their maximum value at ~45°, corresponding to the crystalline axis. Then we plotted the in-plane MR and Kagome net in Supplementary Fig. 14f, where lattice orientation is determined by Raman measurements. One can see that every lobe of MR almost aligns with the symmetric axis of Kagome net.

By fitting the data with Lorenz function, we obtained the precise frequency values of $A_{1g}$ and $E_{2g}$ modes in flake sample and bulk crystal at different angles in Supplementary Fig. 14d. We find that there is no distinguishable difference in the $E_{2g}$ frequency between the flake and bulk samples. It is suggested that both the flake and bulk samples have almost identical in-plane crystal structure and thereby the strain effect from the substrate is completely negligible. However, compared to the bulk crystals, $A_{1g}$ (out-of-plane vibration) mode in flake undergoes a blue shift, indicating that flake sample has a weaker interlayer coupling consistent with the previous studies[17]. That is why flake sample has slightly higher $T_c$ and lower $T_{CDW}$ than the bulk.

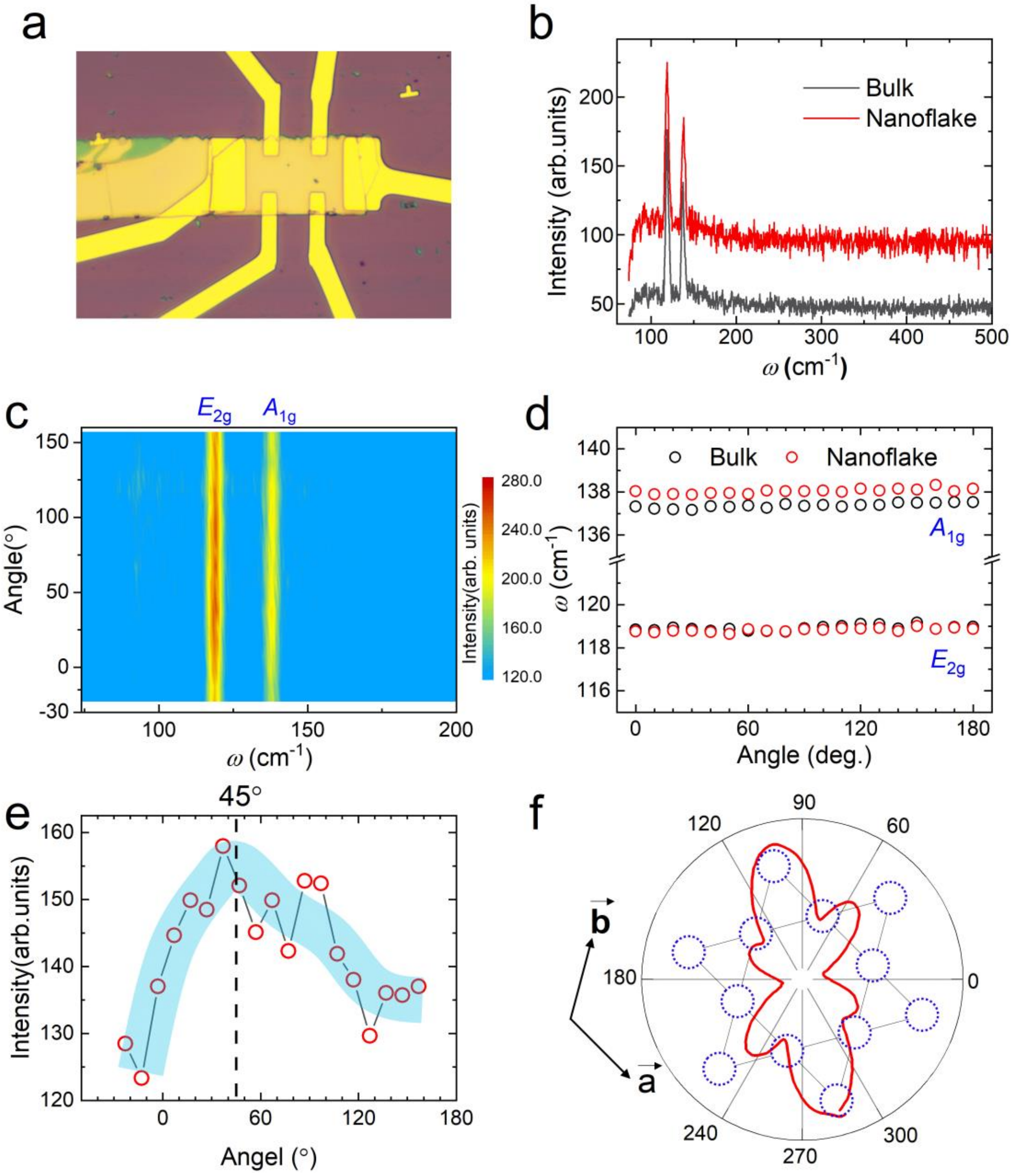


**Supplementary Fig. 14** Raman spectra of $CsV_3Sb_5$ flake and bulk samples. **a,** The optical picture of flake sample with Hall bar electrodes. **b,** Comparison of Raman spectra obtained in $A_{1g}$+$E_{2g}$ symmetry for bulk and flake samples. **c,** Raman scattering intensity measured in parallel polarization with polarization rotated 180° on flake sample. **d,** $A_{1g}$ and $E_{2g}$ phonon frequency as a function of polarization angle for bulk and flake samples. **e,** Polarization angle dependence of $E_{2g}$ phonon intensities in flake sample. **f,** In-plane MR vs. kagome lattice. The orientation of kagome lattice is determined by Raman data in **e**. All the Data were collected at 5 K.

## Supplementary Note 10: Thickness dependence of anisotropic in-plane MR

Our previous studies have shown that the dimensional crossover from 3D to 2D occurs at a thickness of 30 nm in CVS[19]. To investigate the dimensional nature of hidden phase transition, we measured the in-plane MR in a thinner sample with thickness of ~20 nm. The data was plotted in Supplementary Fig. 15. Different from thicker flake samples (≥30nm), we only observed classical Lorentz force MR in the 20 nm sample (termed C2* in our main text), e.g., the MR is always positive, the magnetic field dependence is quadratic, and the minimum MR is along the **I**//**B** configuration (see Supplementary Fig. 15a). This indicates that the hidden order is 3D in nature.

Additionally, given that the substrate strain will be gradually released as the thickness of the samples increases, thinner samples will experience stronger strain effect. That means, in the thinner sample, 1) we did not observe any strain effect in the MR data, and 2) the dimensional crossover[19] destroys the hidden phase, rendering the C2 and C6 components to vanish together with the negative MR. This fully demonstrates that the unusual anisotropic in-plane negative MR observed in our work does not result from the substrate strain effect.

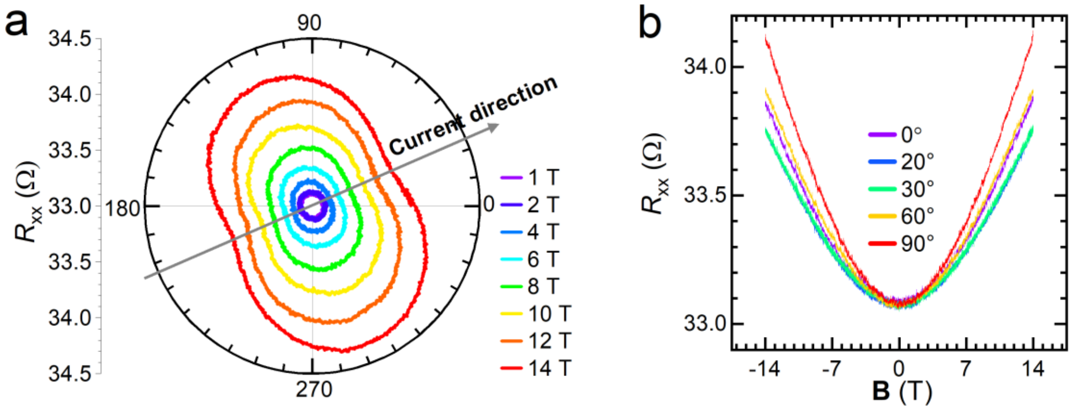


**Supplementary Fig. 15** In-plane MR in $CsV_3Sb_5$ with the thickness of ~20 nm. **a,** Polar plot of the in-plane MR at different magnetic field orientations at a number of magnetic field values from 1 T to 14 T. The current direction is marked in the plot, showing it origin as the Lorentz effect. **b,** The positive, monotonic, and quadratically increasing MR further demonstrates its classical origin.

## Supplementary Note 11: Phenomenological model for anisotropic in-plane MR

To well understand the relationship between anisotropic in-plane MR and hidden order, we propose a simple model in the following:

1) C2 component (nematic order from CDW and/or electronic nematicity). Now we consider the situation where the material become anisotropic due to additional ordering. Assuming the material with a C2 symmetry order and the order direction has an angle of $\alpha$ degrees with respect to the current direction, as shown in Supplementary Fig. 16a. In Supplementary Fig. 16a, we define the current direction to be the *x*-direction, and *y*-direction to be perpendicular to the *x*-direction. It is reasonable to hypothesize that electrons moving perpendicular to the order direction (marked as $v_{x,\perp}$ in Supplementary Fig. 16a) have larger effective mass (band modification) and/or stronger scattering rate (combined impurity effects with band modification) than along the parallel direction (marked as $v_{x,//}$). Here $v_x$ denotes the average velocity of the charge carriers under current **I**. In this case, we only need to consider the effect of magnetic field on $v_{x,\perp}$. Thus the scattering probability $1/\tau$ from C2 order is mainly related to $B$ component along the C2 direction, inducing a MR with C2 symmetry. In terms of band structure modification, the magnetic field creates helical carrier trajectory which increases the weight of transport along the magnetic field direction, i.e., forcing more electrons along the direction along the magnetic field with an effective mass changing with C2 symmetry, creating similar effects to that of the impurities scattering. Therefore, in-plane MR vs. rotating $B$ angle induced by a C2 symmetric order is also of C2 symmetry. In contrast to C2* MR which is defined by the direction of the current, the C2 component would have a direction determined by the C2 nematic order. Supplementary Fig. 16b shows the in-plane MR in two special situations where $\alpha$ = 90 °(upper panel) and 0 °(lower panel) in $CsV_3Sb_5$. When $\alpha$ = 90 °, under a rotating magnetic field with angle $\gamma$ with respect to the *y*-direction, maximum MR is achieved with $\gamma$ = 90 °, while minimum MR is achieved with $\gamma$ = 0 °. Similar situation can be found for $\alpha$ = 0 °.

   Now we use this model to explain the observed C2 symmetry MR of $CsV_3Sb_5$. In kagome metal $CsV_3Sb_5$, the C2 symmetry order has been confirmed to be a $\pi$ phase shift between neighboring layers below $T_{CDW}$ and an electronic nematic order below 35 K. This is consistent with our results in Figure 1c of the main text and Supplementary Fig. 8c, i.e., the C2 symmetry is not determined by current direction, but by the order direction.

2) C4 component (coupling effects). As discussed in the main text, the appearance of the C4 component is a coincident with C2* and C2 components orientated 90° away from each other. Consider finite coupling between the effect of isotropic impurity scattering and the anisotropic effect by C2 nematic orders, $\rho_{xx} \propto 1/\tau_{impurities}\cos(2\gamma) + 1/\tau_{nematic\ order}\cos(2\gamma + 90°) + \delta(impurities, nematic\ order)$, where $1/\tau_{impurities}$ is from impurities scattering, $1/\tau_{nematic\ order}$ is from the effective nematic order scattering including band modification effects, and $\delta(impurities, nematic\ order)$ denotes their coupling term. The coupling term can be simply understood as:

$$\delta(impurities, nematic\ order) \propto \frac{(1/\tau_{impurities})(1/\tau_{nematic\ order})}{(1/\tau_{impurities})+(1/\tau_{nematic\ order})}\cos(2\gamma)\cos(2\gamma+90°)$$

$$\propto \cos(4\gamma)。$$

At low and high fields, $1/\tau_{impurities}$ and $1/\tau_{nematic\ order}$ dominate and providing C2* and C2 signals, respectively. At the intermediate field, the coupling term reaches maximum value and shows a C4 symmetric MR, which is in accord with our results in Figure 2a.

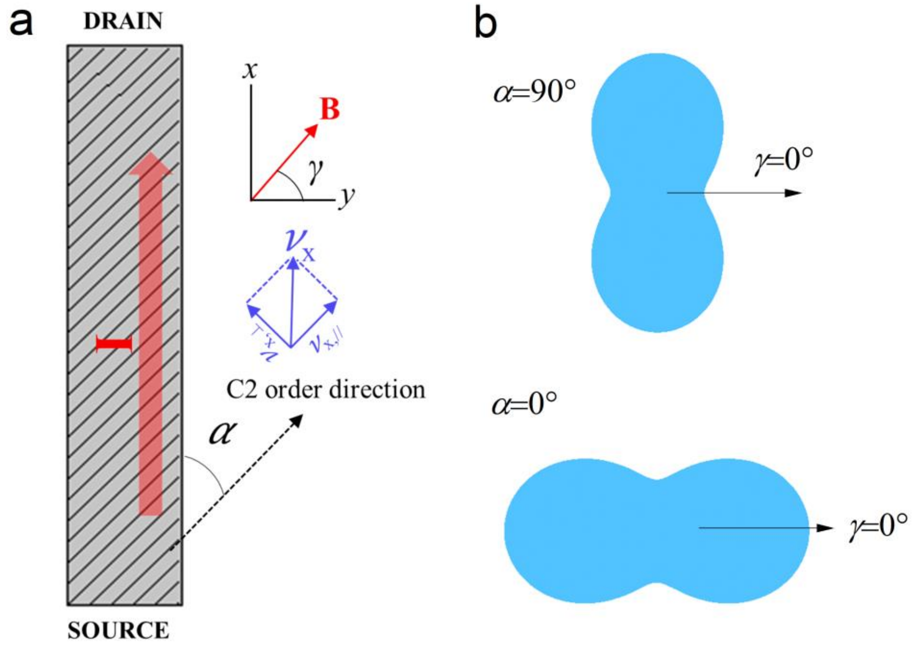


**Supplementary Fig. 16** In-plane MR model. **a,** Schematic diagram of a device. There is a C2 symmetric order in the sample, and its direction forms an angle of $\alpha$ degrees with the x-direction. The rotating **B** is defined by an angle $\gamma$ with respect to the y-direction. **b,** In-plane MR vs. $\gamma$ when $\alpha$ = 90° and 0°.

3) C6 component (hidden order). The C6 component can be understood in ways similar to discussion of the C2 component in 1), which is the key finding in our experiment. If the hidden order has three symmetric axes, it would cause the effective scattering probability vs. **B** to exhibit a six-fold symmetry, providing the C6 component in the in-plane MR. And we have also excluded the possibility of trivial origins of this C6 component, such as from crystal symmetry.

To summarize, the C2 and C6 components of MR are mainly due to the influence of magnetic field changing the effective electron scattering probability, with contribution from both impurity scattering and from band structure modification, as compares to only the impurity scattering from the classical C2* component. Thus, as **B** increases, the C2 and C6 components become more prominent.

**Supplementary Note 12: Out-of-plane angle dependence of negative in-plane MR**

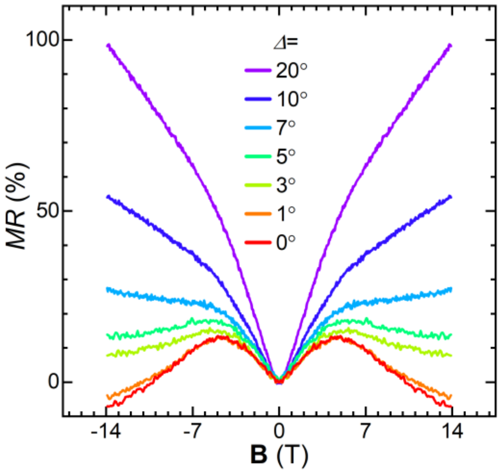


**Supplementary Fig. 17** MR with different angles (Δ) deviation from the *x*-*y* plane. When $\Delta = 0°$, the magnetic field is perpendicular with the current direction. With increasing magnetic field tilted from the *x*-*y* plane, the amplitude of the negative MR significantly decreases. Once the tilting angle is above 5°, the negative MR completely vanishes. This phenomenon can be understood as increasing out-of-plane magnetic field strengthens in-plane orbital current order and thereby suppresses its fluctuations, which is also in accord with the proposed theory of fluctuations of the orbital current order in main text.

**Supplementary Note 13: Negative in-plane MR versus the applied current direction**

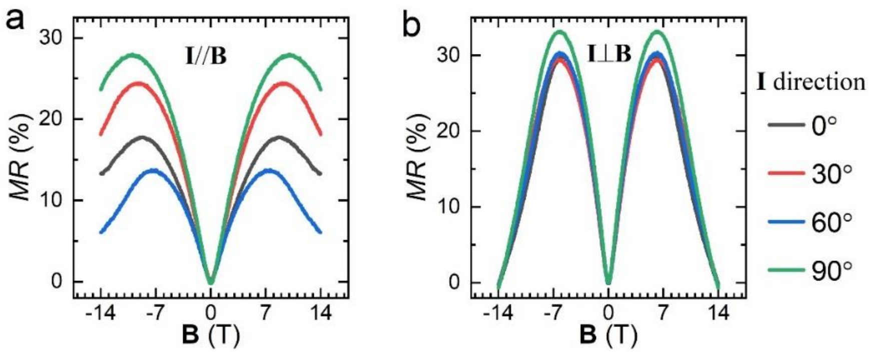


**Supplementary Fig. 18** Negative MR with various current directions. **a,** MR with **I**//**B**; **b,** MR with **I** ⊥**B**. As for **B**⊥**I**, negative MR is hardly affected by the direction of the current in **b**. Interestingly,

negative MR with **B**//**I** configuration changes significantly on the direction of the current in **a**. Generally, MR from conventional electron-impurity scattering in the **B**//**I** configuration is much smaller than that in the **B**⊥**I** configuration. Our data suggested that the negative MR must results from an unconventional scattering, which we link to the hidden order in our work.

**Supplementary references**


1. Fu Y, *et al.* Quantum Transport Evidence of Topological Band Structures of Kagome Superconductor $CsV_3Sb_5$. *Phys Rev Lett* **127**, 207002 (2021).
2. Huang X, *et al.* Three-dimensional Fermi surfaces from charge order in layered $CsV_3Sb_5$. *Phys Rev B* **106**, 064510 (2022).
3. Thopart D, Wahl A, Maignan A, Simon C. Negative in-plane and out-of-plane magnetoresistivities in an optimally doped $Bi_2Sr_2Ca_{0.8}Y_{0.2}Cu_2O_{8+\delta}$ single crystal. *Phys Rev B* **62**, 5378 (2000).
4. Luo H, Wen H-H. Localization of charge carriers in the normal state of underdoped $Bi_{2+x}Sr_{2-x}CuO_{6+\delta}$. *Phys Rev B* **89**, 024506 (2014).
5. Li Q, *et al.* Chiral magnetic effect in $ZrTe_5$. *Nat Phys* **12**, 550-554 (2016).
6. Huang X, *et al.* Observation of the Chiral-Anomaly-Induced Negative Magnetoresistance in 3D Weyl Semimetal TaAs. *Phys Rev X* **5**, 031023 (2015).
7. Li H, *et al.* Negative magnetoresistance in Dirac semimetal $Cd_3As_2$. *Nat Commun* **7**, 1-7 (2016).
8. Hikami S, Larkin AI, Nagaoka Y. Spin-orbit interaction and magnetoresistance in the two dimensional random system. *Prog Theor Phys* **63**, 707-710 (1980).
9. Wang Z-C, *et al.* Giant linear magnetoresistance in half-metallic $Sr_2CrMoO_6$ thin films. *NPJ Quantum Mater* **6**, 53(2021).
10. Narayanan A, *et al.* Linear Magnetoresistance Caused by Mobility Fluctuations inn-Doped $Cd_3As_2$. *Phys Rev Lett* **114**, 117201 (2015).
11. Wu HC, *et al.* Large positive in-plane magnetoresistance induced by localized states at nanodomain boundaries in graphene. *Nat Commun* **8**, 14453 (2017).
12. Pippard AB. *Magnetoresistance in metals*. Cambridge university press (1989).
13. McGuire T, Potter R. Anisotropic magnetoresistance in ferromagnetic 3d alloys. *IEEE Transactions on Magnetics* **11**, 1018-1038 (1975).
14. Liu Q, *et al.* Nontopological origin of the planar Hall effect in the type-II Dirac semimetal $NiTe_2$. *Phys Rev B* **99**, 155119 (2019).
15. Wu J, Bollinger A, He X, Božović I. Spontaneous breaking of rotational symmetry in copper oxide superconductors. *Nature* **547**, 432-435 (2017).
16. Wulferding D, *et al.* Emergent nematicity and intrinsic versus extrinsic electronic scattering processes in the kagome metal $CsV_3Sb_5$. *Phys Rev Research* **4**, 023215 (2022).
17. Song B, *et al.* Anomalous enhancement of charge density wave in kagome superconductor $CsV_3Sb_5$ approaching the 2D limit. *Nat Commun* **14**, 2492(2023).

18. Liu G*, et al.* Observation of anomalous amplitude modes in the kagome metal $CsV_3Sb_5$. *Nat Commun* **13**, 3461 (2022).
19. Wei X*, et al.* Linear nonsaturating magnetoresistance in kagome superconductor $CsV_3Sb_5$ thin flakes. *2D Mater* **10**, 015010 (2022).
